# Direct Intercellular Communications and Cancer: A Snapshot of the Biological Roles of Connexins in Prostate Cancer

**DOI:** 10.3390/cancers11091370

**Published:** 2019-09-14

**Authors:** Catalina Asencio-Barría, Norah Defamie, Juan C. Sáez, Marc Mesnil, Alejandro S. Godoy

**Affiliations:** 1 Departmento de Fisiología, Pontificia Universidad Católica de Chile, Santiago 8331150, Chile; 2 STIM Laboratory, Université de Poitiers, 86073 Poitiers, France; 3 Instituto de Neurociencias, Centro interdisciplinario de Neurociencias, Universidad de Valparaíso, Valparaiso 2340000, Chile; 4 Centro de Biología Celular y Biomedicina (CEBICEM), Universidad San Sebastián, Santiago 7510157, Chile; 5 Department of Urology, Roswell Park Comprehensive Cancer Center, Buffalo, NY 14263, USA

**Keywords:** intercellular communication, connexins, cancer, prostate cancer

## Abstract

Tissue homeostasis is the result of a complex intercellular network controlling the behavior of every cell for the survival of the whole organism. In mammalian tissues, cells do communicate via diverse long- and short-range communication mechanisms. While long-range communication involves hormones through blood circulation and neural transmission, short-range communication mechanisms include either paracrine diffusible factors or direct interactions (e.g., gap junctions, intercellular bridges and tunneling nanotubes) or a mixture of both (e.g., exosomes). Tumor growth represents an alteration of tissue homeostasis and could be the consequence of intercellular network disruption. In this network, direct short-range intercellular communication seems to be particularly involved. The first type of these intercellular communications thought to be involved in cancer progression were gap junctions and their protein subunits, the connexins. From these studies came the general assumption that global decreased connexin expression is correlated to tumor progression and increased cell proliferation. However, this assumption appeared more complicated by the fact that connexins may act also as pro-tumorigenic. Then, the concept that direct intercellular communication could be involved in cancer has been expanded to include new forms of intercellular communication such as tunneling nanotubes (TNTs) and exosomes. TNTs are intercellular bridges that allow free exchange of small molecules or even mitochondria depending on the presence of gap junctions. The majority of current research shows that such exchanges promote cancer progression by increasing resistance to hypoxia and chemotherapy. If exosomes are also involved in these mechanisms, more studies are needed to understand their precise role. Prostate cancer (PCa) represents a type of malignancy with one of the highest incidence rates worldwide. The precise role of these types of direct short-range intercellular communication has been considered in the progression of PCa. However, even though data are in favor of connexins playing a key role in PCa progression, a clear understanding of the role of TNTs and exosomes is needed to define their precise role in this malignancy. This review article summarizes the current view of the main mechanisms involved in short-range intercellular communication and their implications in cancer and delves into the biological, predictive and therapeutic role of connexins in PCa.

## 1. Introduction

Tissue homeostasis is the result of a complex intercellular network controlling the behavior of every cell to ensure correct function of the organs they belong to and survival of the organism. Accordingly, in mammalian tissues, cells communicate via diverse long- and short-range communication mechanisms. While long-range communication involves the action of hormones through general blood circulation and neural transmission, short-range communication includes either paracrine diffusible factors (e.g., growth factors and cytokines) or direct interactions (e.g., gap junctions, intercellular bridges and tunneling nanotubes) or a mixture of both (e.g., exosomes).

Tumor growth is a disruption of tissue homeostasis and has been long known that it could be the consequence of intercellular network disruption or, at least, dysfunction of some of its components. In this intercellular network, extensive studies have shown that it is mostly short-range intercellular communication that is involved in this characteristic disruption of tissue homeostasis. Although special attention has been given to the paracrine component of such a network, which was found as a result of the discovery of oncogenes/tumor suppressor genes that control cancer cell growth, an even earlier attention was given to the role of intercellular communication mediated by direct cell-to-cell interactions in cancer development.

## 2. Cancer Involvement of Direct Intercellular Communications

### 2.1. Gap Junctions and Cancer

Over 50 years ago, direct cell-to-cell communication received attention in cancer research through the study of gap junctions. More specifically, it was about the electrical coupling present between normal hepatocytes and its loss in liver cancer cells [1]. The finding began a constant accumulation of data supporting this initial observation in cancer cells from various tissues and from diverse mammalian species. Initially, the lack of electrical coupling (or dye transfer) was associated with the absence of gap junctions between cancer cells. This was demonstrated by using electron microscopy which showed that gap junctions were absent in invasive or metastatic cells from cervical squamous carcinomas [2]. After cloning the protein subunits of gap junction channels, previously termed connexins (Cxs), it became clear that Cxs are not functional in tumor cells because of lack of expression or aberrant subcellular localization. This observation suggested that tumor cells can escape from normal cell control because of the lack of functional gap junctions between them. This idea was confirmed by experiments showing that chemical re-induction of gap-junctional intercellular communication (GJIC) between cancer cells and normal cells was sufficient to decrease their growth [3]. More generally, reduced cell proliferation was observed after re-inducing Cx expression through Cx cDNA transfection, supporting the hypothesis that Cxs are putative tumor suppressors themselves [4]. The tumor suppressor role of Cxs or GJIC was reinforced by the fact that non-genotoxic chemicals, known as tumor promoters (e.g., phorbol esters), as well as some tumor viruses (e.g., avian sarcoma virus), reduce GJIC [5,6,7]. Later, this apparently clear picture of Cxs/gap junctions appearing as tumor suppressors became confused by a series of observations. The first was that the recovery of GJIC is not always associated with decreased cell proliferation but depends on the Cx type that is re-expressed in cancer cells [8]. The second observation was that contrary to classical tumor suppressors, Cx gene mutations are rare and those which have been described in some types of cancer do not appear as driver mutations [9]. This places Cxs among Class II tumor suppressors, acting through aberrant expression. This is contrary to classical Class I tumor suppressors whose effect is the consequence of defective gene mutations [10]. If not caused by Cx gene mutations, loss of GJIC in cancer cells was shown to be the consequence of events targeting Cxs and known to modulate their function at the post-translational level such as phosphorylation or cytosolic Ca^2+^ concentration [11,12]. Among these events, phosphorylation has been studied extensively and shown that Cx43 function is tightly controlled by kinases and phosphatases [13]. Multiple tumor promoters and oncogenes have been shown to inhibit Cx43-mediated GJIC through specific phosphorylation of this connexin. This is the case of 12-O-tetradecanoyl-13-acetate (TPA) acting through protein kinase C (PKC) and extracellular signal-regulated kinase ERK activation [14,15] or the activated proto-oncogene tyrosine-protein kinase Src through tyrosine phosphorylation [16].

There are only a few examples of cells from advanced cancers that have been shown to still express Cxs. These observations appear as exceptions and they do not seem to contradict the general assumption that decreased Cx expression was correlated to tumor progression and increased cell proliferation. This was the case until a new study showed that Cxs that were still expressed in cancer cells could be pro-tumorigenic because they promoted cell migration [17]. Such observations have been collected during the last decade and support a more consolidated hypothesis that Cxs could act as both inhibitors of cell proliferation and inducers of cell migration and invasion [18]. Cxs could be an important factor in what was called the “grow or go” behavior of cancer cells. They would be able to decrease growth of cancer cells when present by activating their migration out of the tumor core. Consequently, some efforts have been made to use Cx identification and abundance in some tissues as part of diagnosis, which requires further study for validation. This would be important for using Cxs to reduce or eliminate cancer cells through the so-called bystander effect, in which radiation or cisplatin toxicity can be propagated via gap junctions among cancer cells [19].

### 2.2. Intercellular Bridges, Tunneling Nanotubes and Cancer

Direct transfer of molecules and organelles has been described to occur through intercellular bridges. These bridge structures result from incomplete cytokinesis and contain microtubules. They facilitate transfer of cytoplasmic content including vesicles mainly between normal germ cells and some cancer cells that could be deficient in GJIC. Intercellular bridges can be found between pairs or group of cells and can range from 100 to 200 nm for thin extensions and 1 to 5 µm for wider structures with length between a few microns and at least 50 to 100 µm containing alpha-tubulin bundles. It is believed that intercellular bridges allow direct transfer of cytoplasmic molecules and organelles helping to synchronize cell metabolism and state of differentiation [20]. The role of such structures in cancer has not been well illustrated but it could have an influence on cancer phenotype since fusion between cancer cells and normal cells was shown to lead to a normal phenotype [21] suggesting that cancer phenotype is not dominant. To our knowledge, this assumption has not been explored and confirmed further. 

More recently, intercellular bridges, called tunneling nanotubes (TNTs) have been described as long cytoplasmic extensions (up to 300 µm length; 50–800 nm diameter) that enable long-range, directed communication between connected cells [22]. Time-lapsed imaging studies suggest that TNTs are formed as an actin-driven protrusion outgrowth [23,24,25] similar to filopodia-like protrusion containing F-actin, in which fascin, an actin filament bundling protein, has been identified [26]. After extension, the tip of the filopodia-like protrusion contacts the target cell directly (or through adhesion molecules) and may fuse with the receiving cell [25]. These structures have been observed as open ended or gap junction/connexin-containing protrusions. It has been proposed that TNTs could also be the consequence of cells moving apart after having been fused temporarily and partly (i.e., immune synapse) stretching out the fused plasma membranes [23,24,25,27,28,29]. Structures like "mitotic nanotubes" would belong to this last case since they are formed by mitotic cells with adjacent cells during rounding. This formation may explain the presence of gap junctions in some instances since Cx43 can localize in these intercellular bridges during mitosis [30].

Actin cytoskeleton is present in all kinds of TNTs and its polymerization plays an important role in their formation [25,31,32]. In some cells (e.g., macrophages), TNTs have been shown to contain also microtubules organized in bundles parallel to the major axis [33]. These differences of organizations and/or composition may be linked to different functions of TNTs as it has been observed in normal and cancer urothelial cells in which the fact that microtubules helically enwrap intermediate filaments may provide particular elasticity and rigidity to these structures [34].

Whatever their structure, TNTs seem to be involved in the intercellular transport of cytoplasm and vesicles between normal cells and cancer cells, and also between cancer cells and stroma cells. Depending if they are open-ended or not with the presence of gap junctions, TNTs have been shown to allow other types of intercellular transports such as mitochondria, electrical signals and small molecules [35]. If the precise molecular mechanisms involved in the formation of TNTs are not completely understood, stimuli leading to this process should be identified. In the cancer context, such stimuli are related to stress situations such as hypoxia and chemotherapy. 

Hypoxic conditions stimulate TNT formation in chemoresistant ovarian cancer cells through the mammalian target of rapamycin (mTOR) pathway whose inhibition leads to suppression of these structures [36]. Interestingly, TNT formation can be detected between chemosensitive and chemoresistant ovarian cancer cells [36]. In this particular context, the role of such TNT formation is not known but could provide mitochondrial transfer that has been frequently observed through TNTs. Such a transfer could help metabolic adjustments which then favor the tumor development helping cancer cells to escape hypoxic conditions. For instance, acute myeloid leukemia (AML) relies on oxidative phosphorylation to generate adenosine triphosphate. The reliance of multiple myeloma cells on oxidative phosphorylation is caused by intercellular mitochondrial transfer to multiple myeloma cells from neighboring non-malignant bone marrow stromal cells through TNTs. This phenomenon appears as a response to hypoxia inside the tumor and is susceptible to decreased survival of the tumor host [37,38]. Interestingly, when the mitochondrial transfer is decreased between mesenchymal stem cells and Jurkat cells, it increases chemotherapy-induced cell death [39]. Mitochondrial transfer between bone marrow microenvironment and leukemic cells thus appears as a new mechanism of drug resistance [40]. Therefore, mitochondrial transfer is associated with increased cancer cell survival and chemoresistance. It also seems to favor invasiveness, as it was observed in bladder cancer cells [41]. Supply of mitochondria to cancer cells can be supported by endothelial cells through TNTs [42]. Here also, TNT-transferred mitochondria were shown to change the metabolism of recipient cells favoring the emergence of chemoresistance in cancer cells [42]. These results are in phase with the fact that changes in metabolic pathways, including glycolysis, pentose phosphate and lipid metabolism, are linked to cancer cell resistance to therapy [43].

Thus, hypoxia resistance and chemoresistance seem to be increased through TNTs contributing between malignant and stromal cells. Such connections can lead to changes in gene regulation participating in cancer cell progression [44]. The molecular consequences of such heterocellular connections are not well known. However, a number of concrete examples have started to emerge, such as the transferrin receptor that is transferred from cancer cells to fibroblasts. This transfer involves the function of the small guanosine triphosphatase (GTPase) Rab8, which is colocalized with the transferrin receptor in the TNTs and is cotransferred to acceptor cells [45]. These types of connections were found to be initiated from astrocytes towards C6 glioma cells; a phenomenon dependent on the tumor protein p53 and able to reduce the proliferation of C6 glioma cells [46]. Macrophages may also contribute to TNT formation among cancer cells via the secretion of unidentified factors [47]. TNTs could also be involved in tumor angiogenesis originating from pericytes that are looking to actively explore the microenvironment and connecting with targeted vessels through other pericytes or endothelial cells [48]. Alternatively they may also play a role in migration because TNTs can be formed by migrating cancer cells and extend during migration [26].

Chemotherapeutics are a stress capable of inducing TNT formation in cancer cells which may also participate in chemoresistance. For instance, doxorubicin stimulates formation of TNTs in pancreatic cancer cells which facilitates the intercellular redistribution of the drug between connected cells in vitro. Moreover, it was observed that TNT formation was upregulated in aggressive forms of pancreatic carcinoma and was further stimulated after chemotherapy exposure acting as a novel method for drug efflux [49]. Despite possible diffusion of the chemotherapeutic agent between cancer cells, it has been observed that TNTs could increase chemoresistance in cancer cells by permitting P-glycoprotein (encoded by the multidrug resistance gene-1, MDR1) transfer between the Michigan Cancer Foundation-7 (MCF-7) breast cancer cells, a phenomenon which can also occur through exosomes [50].

Interestingly, TNTs and exosomes (or other extracellular vesicles) seem to be associated at different levels. For instance, treatment with macrophage conditioned medium not only enhances TNT formation between cells but also stimulates the release of cytoplasmic fragments, referred to as microplasts, from MCF-7 cells through formation of TNT-like structures [47]. Mitochondria, vesicles and cytoplasm can be transferred from cell body to microplasts through connecting TNTs. The microplasts can also be resorbed into the cell body by retraction of the connecting TNTs [47]. Moreover, mesothelioma cells co-cultured with exogenous mesothelioma-derived exosomes form more TNTs than cells cultured without exosomes which are localized predominantly at the base of and within TNTs, in addition to the extracellular environment. Time-lapse microscopic imaging demonstrated uptake of tumor exosomes by TNTs, which facilitated intercellular transfer of these exosomes between connected cells. Mesothelioma cells connected via TNTs were also enriched for lipid rafts. This study provided supportive evidence of exosomes as potential chemotactic stimuli for TNT formation, and lipid raft formation as a potential biomarker for TNT-forming cells [51]. The link between TNTs and exosomes may be mediated by the Ras association domain-containing protein 1 (RASSF1A), a key-regulator of cytoskeleton. When the inactivation of RhoB guanine nucleotide exchange factor, GEF-H1, is induced by RASSF1A silencing, it leads to accumulation of the Ras related protein 11 (Rab11) and subsequent exosome releasing, which in turn contribute to TNTs formation [52].

To summarize, TNTs may play an important role in critical aspects of cancer including cellular invasion, metastasis, and emergence of chemotherapy drug resistance. Through these different aspects, they probably act also on intratumor heterogeneity [44,53]. Therefore, it seems that preventing TNT formation could be a potential therapeutic possibility that would affect tumor metabolism and progression [38].

### 2.3. Exosomes and Cancer

Another cell-to-cell communication mechanism that has been studied in cancer is exosomes. They are membrane vesicles of endocytic origin ranging from 30 to 150 nm in diameter, released by almost all, if not all, cell types. They contain proteins, lipids and nucleic acids, which can be transferred locally or even systemically between tumor cells and to other cells such as fibroblasts, endothelial cells, progenitor cells, and cells of the immune system. In addition, Cx43 forming hexameric channels have been found at the membrane of exosomes isolated from the extracellular medium of cultured cells or different body fluids. Since inhibition of Cx43 channels drastically reduced transfer of exosome content to cells it was proposed that they modulate the interaction between exosomes and acceptor cells, facilitating the release of exosomal cargo into cells [54]. 

Recently, using bioinformatics analysis it was proposed that RNA- and DNA-binding motifs found in Cx43 and Cx26 sequences might be important to consider for future studies on transfer of genetic information through extracellular vesicles [55].

Intercellular bridges, tunneling nanotubes and exosomes have been proposed to mediate transfer of nucleic acids relevant in cancer metastasis and thus could be crucial targets to reduce or completely prevent this critical event in cancer development. On the other hand, hemichannels and gap junction channels between cancer cells and endothelial cells have been shown to mediate signaling transfer required for cellular extravasation and thus they could participate in propagation of metastatic cells.

## 3. Prostate Cancer, an Example of the Involvement of Direct Intercellular Communications in Cancer

### 3.1. Biological and Clinical Aspects of Prostate Cancer

Prostate cancer (PCa) is one of the malignancies with the highest incidence and represents the second leading cause of cancer-related death in American and European men [56,57,58]. Huggins and Hodges reported in 1941 [59] that growth of PCa depended on androgens, and this conceptual break-through led to the development of androgen deprivation therapy (ADT). It consists of either surgical (orchiectomy) or chemical castration (combination of luteinizing hormone-releasing analogs or antagonists with anti-androgen/androgen receptor-inhibitors) and has represented the standard treatment for locally advanced or metastatic PCa for over 70 years. Circulating testosterone levels are dramatically reduced by ADT, producing reduction in serum prostate-specific antigen and clinical response in most patients. However, despite significant initial response, after 24–36 months the disease progresses and becomes resistant to ADT, with acquisition of a “castration-resistant phenotype (CRPC)” [60,61,62]. A universal mechanism by which PCa evolves to a more aggressive, and eventually lethal, castration-resistant phenotype has not yet been identified [63]. Despite the technological advances achieved over the last decade in the treatment of patients with metastatic CRPC, average survival still remains in three years [62,64]. Both ADT and new generation hormonal therapies, such as abiraterone and enzalutamide, are effective and usually well-tolerated treatments for PCa patients [61]. However, given the long duration of these treatment and their potential side effects on patient’s functional status and quality of life, the clinicians face a particular challenge to prevent or delay the onset of metastatic disease and the resulting mortality, taking into account the negative impact that treatment may have on patient’s quality of life, and at the same time, avoiding over-treatment of PCa patients that have a low risk of clinical progression [61,65]. It is necessary to identify new biomarkers/prognostic factors that can distinguish between clinically significant and less aggressive tumors in order to improve management of PCa patients as well as new potential therapeutic targets to counteract advanced/metastatic PCa.

### 3.2. Gap Junctions and Prostate Cancer

#### 3.2.1. Gap Junctional Intercellular Communication in Benign and Malignant Human Prostate Epithelial Cells

Ample evidence has shown that dysregulation of signaling pathways involved on intercellular communication mediated by direct cell-to-cell contact participates in carcinogenesis and tumor progression [9,17,66,67]. The dysfunction of structures that directly link cytoplasmic compartments of adjacent cells, such as gap junctions, composed of Cxs, have been clearly implicated in PCa development and progression [68,69]. In the prostatic epithelium, previous reports [68,70,71] have identified the presence of at least three Cx isoforms (Cx26, Cx32 and Cx43) exhibiting specific expression patterns. Cx43 was predominantly observed in undifferentiated progenitor and basal epithelial cells, whereas Cx32 was present in luminal secretory epithelial cells with an expression profile linked to the differentiation status of the secretory cells [72,73,74]. On the contrary, homotypic gap junctions composed of Cx26 have been detected between adjacent basal and luminal epithelial cells, which could allow communication between these two cellular compartments, since the connexons composed of Cx32 and Cx43 do not form functional heterotypic channels [75,76].

Aberrant (increased or decreased) expression of various Cxs has been observed in malignant tissues and cancer cell lines from prostate [77,78,79]. In PCa, in a limited set of paired benign and malignant human prostate tissue specimens, a reduction in the steady state level of Cx43 was shown in neoplastic tissues when compared to benign human prostate tissues [80]. An immunohistochemical study [81] demonstrated that Cx32 expression was significantly decreased in PCa when compared to benign human prostate clinical specimens, with a severe loss of Cx32 expression in poorly differentiated PCa specimens [81]. Analysis of the pattern of distribution of Cx32 and Cx43 in human prostate tumor with different histological grades, showed that the immunostaining for Cx32 and Cx43 was poorly detected in the majority of epithelial cells from undifferentiated prostate tumors [81]. On the other hand, in poorly differentiated tumors, Cx32 and Cx43 immunostaining was predominately detected at the cytoplasmic level of cancer epithelial cells [81]. It was reported that the level of expression of Cx43 in PCa showed a negative correlation with established features indicative of worse prognosis such as follow-up time and preoperative PSA. In this study, univariate and multivariate analyses indicated that a decreased in Cx43 expression was found to be a significant predictor of biochemical recurrence free-survival (BFS) [82]. In addition, a significant reduction or loss of Cx43 expression in PCa tissues was associated with advanced clinico-pathological features and poor BFS of patients after radical prostatectomy [83]. In vitro studies [84] demonstrated that ALVA-31, ALVA-41, ALVA-55, and PC-3 cell lines only expressed Cx43, whereas human prostate epithelial cells established from benign donors expressed Cx32 and Cx40. Other studies [85,86] demonstrated that LNCaP and PC-3 cell lines not only expressed Cx43 but also Cx26 and Cx45. High metastatic potential of PCa cell lines correlated with an increase in Cx26 and Cx43 levels and a decreased in Cx32 level. A qRT-PCR analysis performed in our laboratory on LNCaP, LNCaP-C4-2, DU-145 and PC-3 cell lines indicated that there was positive and negative association between the aggressiveness of the cell lines and the degree of expression of Cx26 and Cx32, respectively, but no clear association was observed in the case of Cx43 (unpublished data). Together, these observations indicate that levels of expression of Cxs in in vitro models of PCa are still controversial. However, at the tissue level, for the majority of Cxs, a negative association between level of expression and disease progression/aggressiveness was observed. The differences obtained using in vitro models of PCa could be explained, at least in part, by the different types of analyses used in each study.

#### 3.2.2. Hormonal Regulation of Gap Junctional Intercellular Communication in Prostate Cancer Epithelial Cells

Functional studies using in vitro models support the notion that GJIC is impaired in neoplastic cells. In vitro studies in human prostate cell lines [84] indicated that GJIC was either reduced or not detected in malignant compared to benign human prostate epithelial cells. Additional studies using benign and malignant human prostate tissues indicated that Cx43 immunoreactivity was localized at cell-to-cell boundaries in benign but not in malignant human clinical specimens, which suggests the presence of gap junctional plaques in benign but not in malignant human prostate epithelial cells [87]. Over-expression of Cx43 and Cx32 in the PC-3 cell line resulted in the intracellular accumulation of Cxs due to a defective trafficking because of a low expression of α-catenin, a cadherin-associated protein that triggers trafficking and assembly of Cx43 and Cx32 into gap junctions [88]. Interestingly, treatment of PCa cell lines with forskolin enhanced functional activity of Cxs, which suggests that GJIC may be regulated by hormones that work via cAMP-dependent signal transduction pathway [84]. Previous reports showed that androgens regulate formation and degradation of GJIC. Androgens enhanced the formation of GJIC composed of Cx32 in LNCaP cells by facilitating its trafficking from the ER to the cell surface [89]. Activation of androgen receptor (AR) pathway in LNCaP cells was observed to decrease Cx43 expression at the plasma membrane level, which suggests that AR may be an upstream regulator of Cx43 assembly [90]. These reports indicated that activation of AR pathway in PCa cells could represent a potential mechanism that underlies functional regulation of Cxs in PCa cells.

#### 3.2.3. Biological Effects of Connexins in Prostate Cancer Epithelial Cells

GJIC may be important in regulating cell growth and it is suggested that loss of Cx function contributes to carcinogenesis by deregulating the balance between cell proliferation and cell differentiation [91]. It was shown that control of proliferation and differentiation of epithelial cells from prostate tumors may depend on the appropriate assembly of Cx32 and Cx43 into gap junctions [74]. Further studies, however, showed that GJIC is not required for growth of LNCaP and PC-3 cell lines [86]. Heptanol and AGA-gap junction inhibitors did not affect cell proliferation of C4-2B and PC-3M cell lines [85]. However, increased Cx43 expression by retroviral infection in PC-3 cell line was correlated with a reduction in proliferation of these cells [92]. Retinoids—the natural or synthetic derivatives of vitamin A—and vitamin D_3_ have been shown to modulate growth of several PCa cell lines [93,94]. It was found that over-expression and functionality of Cx32 accentuated the growth inhibitory effect of two metabolites of vitamin A and an active hormonal form of vitamin D_3_ in LNCaP cells [93,94]. On the other hand, it was shown that Cx43 expression can greatly increase the sensitivity of LNCaP cells to agents, such as the tumor necrosis factor alpha (TNFα), TNF-related apoptosis-inducing ligand (TRAIL) and anti-Fas antibodies, that induce apoptosis [95]. Over-expression of Cx43 in DU145 and PC-3 cell lines enhanced pro-apoptotic effects induced by chemopreventive and chemotherapeutic agents such as se-methylselenocysteine (MSC) and docetaxel (DTX) [96,97], indicating that Cxs have the ability to modulate PCa cell growth by affecting the balance between cell proliferation and apoptosis.

The paradigm of cancer-suppressive Cx function has been challenged by the observation that Cxs could be re-expressed and have an active role in cancer cell dissemination during the late stages of tumor progression [17,66,92,98,99,100]. Consequently, as observed in several other types of solid tumors, it has been proposed that Cxs could have state-specific functions in the promotion and progression of PCa. An increase of Cx43 expression was correlated with a reduction of adhesion and invasion capacities in PC-3 cells, while Cx43 enhanced cell aggressiveness in LNCaP cells [92]. Despite in vivo and in vitro studies which indicated a lower expression of Cxs in malignant compared to benign human prostate epithelial cells, other reports have found evidence that Cx43 and Cx26 expression were positively correlated with an increase in malignancy of PCa cell lines [85,86]. Treatment with GJIC inhibitors significantly reduced cell migration and invasion through Matrigel in the metastatic LNCaP-C4-2B and PC-3M cell lines [85,86]. This observation was also supported by *in vitro* studies [85] indicating a direct association of Cx26 and focal adhesion kinase (FAK) in PCa cells. On the other hand, suppression of Cx43 expression in PC-3 cells using shRNA inhibited migration and invasion capacities as determined using wound healing and transwell-invasion assays, respectively [86]. It was proposed that selective transmigration of PCa cells, which express high levels of Cx43, may be crucial for the leading front formation during cancer invasion [101]. All together these reports suggest that Cxs may differentially affect the biology of cancer cells and can selectively promote progression of PCa cells in vitro and in vivo.

#### 3.2.4. Connexins in Prostate Cancer Stroma

Cancer is a disease that alters/results from complex interactions between the cancer epithelial cells and their surrounding stromal compartment in which the cancer cells live, which is called the tumor microenvironment (TME) [102]. TME includes multiple different types of non-malignant cells, such as activated fibroblasts, infiltrating macrophages and other immune cells, as well as the tumor microvasculature composed of endothelial cells and pericytes [103]. Cancer progression has been recognized as the product of an evolving communication between cancer cells and their TME [104,105,106]. It has been established that this communication can determine the phenotype of the tumor [104]. In the majority of cancers, TME exhibited an activated phenotype (reactive stroma) composed of a myofibroblast/fibroblast mixture, with a significant decrease of fully differentiated smooth muscle cells, increased extracellular matrix remodeling, increased protease activity and the influx of inflammatory cells, and increased angiogenesis. These changes lead to aberrant growth and morphologic transformation of the stromal tissue, which favor progression of cancer cells [106].

GJIC between cancer cells and their surrounding stroma cells has been described in several tumor types, which include human gliomas and glioblastomas [107,108]. GJIC between PCa cells and their stromal cells has been poorly characterized. Extensive coupling was observed between the highly metastatic rat prostate cancer cell line, MAT-LyLu, and human benign fibroblasts, and these heterotypic contacts were able to stimulate migration of MAT-LyLu cells [109]. On the other hand, it was shown that Cx43 mediated an intercellular signaling that locally activated endothelial cells and augmented the efficiency of PCa cell diapedesis [110]. A recent study found that DU145, but not PC-3, cell line could establish GJIC with endothelial cells. In this study, expression of Cx43 in PCa cells was able to increase the expression of Cx43 in endothelial cells. Up-regulation of endothelial Cx43 was observed during the diapedesis of DU-145 and MAT-LyLu cells and this process was independent of GJIC and dependent of ERK1/2 signaling in endothelial cells [111]. All this evidence suggests that Cxs could play an important role in the communication between PCa cells and their cellular microenvironment, and that these interactions can modulate migration and invasion processes of cancer cells. These interactions may even play a role in PCa metastasis which preferentially target bone tissues. Reintroduction of Cx43 into the poorly metastatic PCa cell line, LNCaP, led to a more aggressive phenotype of those cells and increased bone metastasis in mice [92]. The molecular aspects of this phenomenon are not known yet but could be the consequence of the ability of Cx43 to mediate GJIC between PCa cells and osteoblasts as observed in vitro [71,92]. Despite these interesting findings, more studies are required to determine the extent of the role of Cxs in the biology of PCa cells and to identify and functionally characterize the specific isoforms of Cxs involve in these processes.

### 3.3. Tunneling Nanotubes (TNTs) and Exosomes in Prostate Cancer

So far, the role of TNT formation in PCa has been poorly studied. Kretschmer et al. [112] recently characterized TNT formation in PCa as a potential mechanism for stress adaption and survival. Interestingly, androgen receptor blockade and metabolic stress induce TNTs formation in PCa cells, but not in normal prostatic epithelial cells suggesting that formation of TNTs represents adaptive response specifically coopted by PCa cells to survive therapeutic stress. Moreover, in this study, the authors defined a series of events that involve stress-induced expression of chaperones, clusterin and the Y box binding protein 1 (YB-1) and AR variants, phophoinositide 3-kinase/protein kinase B (PI3K/AKT) signaling, actin remodeling and TNT-mediated intercellular communication, which in conjunction permit cell survival. Remarkably, this work highlights the relevance of TNTs formation as a potential cellular mechanism for ADT treatment resistance in PCa.

In PCa, it has been suggested that cross-talk between tumor epithelial and stromal cells through mRNA- and/or protein-containing exosomes alter PCa cell behavior [113]. Within tumor microenvironment, exosomes can be secreted not only by PCa cells, but also, by carcinoma-associated fibroblasts (CAF), immune cells and endothelial cells [114]. PCa cell lines-derived exosomes can induce transformation of fibroblast to myofibroblast through the activation of the transforming growth factor beta (TGF-β)/SMAD signaling [115,116]. In fact, fibroblast differentiation by TGF-beta from PCa cells was abolished after eliminating exosome secretions [113]. On the contrary, carcinoma associated fibroblasts (CAFs)-derived exosomes can transfer miRNA into neighboring cancer epithelial cells causing further growth of PCa cells [117,118,119]. In addition, exosomes release by PCa cells can modulate the behavior of endothelial cells, which promotes angiogenesis [119]. For instance, exosomes from PCa cells transfer sphingomyelin and cluster of differentiation 147 (CD147) into endothelial cells to support neovascularization [120]. Exosomes released by PCa cells alter immune cells (natural killer and CD8+ T cells), which promote immune suppression and tumor cell escape. Also, PCa cell-derived exosomes can induce an inflammatory response in stromal cells which changes the secretory pattern of exosomes released by CAFs to enhance cancer cell proliferation [113,115,121]. In summary, exosomes released by PCa epithelial cells, and their tumor microenvironment, can synergize to promote PCa growth and progression.

## 4. Conclusions

As it has become more evident for all carcinomas including PCa, there is a complex relationship between their development and state of progression and the level of expression and potential biological roles of Cxs. Most of malignant human prostate tissue specimens showed a drastic reduction and dysfunctional GJIC, primarily due to a defective trafficking of Cx32 and Cx43 into gap junctions. Nevertheless, in vitro studies using cell lines suggested that Cxs could be causally involved in promoting progression of PCa cells. Interestingly, most of these studies have focused on the biological effects of Cx43 on cell migration and invasion. Additional in vitro studies are necessary to elucidate the precise molecular mechanism through which these effects are carried out. Functionality of other subtypes of Cxs, such as Cx26, Cx32 and Cx46, in these biological processes could help to unravel the complex, and still controversial, role of Cxs in PCa. A key step in the progression and metastatic process of PCa corresponds to the interaction between PCa cells and the adjacent cells that comprise their TME. Even though the heterocellular communication process has only been studied between PCa cells and fibroblasts and PCa cells and endothelial cells, the data is highly promising since presence of Cxs in non-malignant cells, especially Cx43, has been shown to modulate PCa cell migration and invasion through GJIC-dependent and -independent processes. In this regard, it would be of great interest to verify whether communication between PCa cells and non-malignant TME cells through gap junctions allows cancer cells to acquire a more aggressive phenotype in vitro and in vivo. In summary, further studies on the specific role of Cxs in the biology of PCa cells as well as the contribution of these proteins in the pro-tumorigenic promoting effects of the TME cells could provide new potential biomarkers and or therapeutic target that could help to counteract PCa. This is especially important in advanced/metastatic stages, since current treatments for this disease produce mostly non-curative responses. In addition, efforts should be focused to understand how other types of intercellular communication such as TNTs and exosomes are involved in PCa progression.
